# BioTM Buzz (Volume 3, Issue 1)

**DOI:** 10.1002/btm2.10087

**Published:** 2018-01-19

**Authors:** Aaron C. Anselmo

**Affiliations:** ^1^ Division of Pharmacoengineering and Molecular Pharmaceutics, Eshelman School of Pharmacy University of North Carolina at Chapel Hill Chapel Hill North Carolina 27599 United States

## Volume 3, Issue 1

### Rapid‐forming keratin‐based hydrogels

Protein‐based biomaterials are widely utilized as scaffolds for tissue engineering applications given their advantages related to biocompatibility and controllable degradation via enzymes. However, issues related to synthesis and manufacturing have limited their clinical use. In this issue of Bioengineering & Translational Medicine, engineers from Harvard and Northeastern Universities detail a method to synthesize kertain‐PEG hydrogels that form when exposed to visible light. Keratin is an essential structural protein and is the predominant component in tissues such as our hair, nails, and skin and thus represents a key protein of interest. The authors show that by utilizing the cysteine rich residues naturally present in keratin, click‐chemistry between the thiol‐norbene groups can be used to form the gels in under one minute. The gels can be tuned to exhibit a variety of mechanical (∼2 kPa to ∼45 kPa compressive modulus) and chemical (complete degradation as fast as 3 hours up to > 5 hours) properties. The scaffolds exhibited the ability to facilitate attachment, spreading, and growth of fibroblast cells in both 2D cell culture and 3D cell encapsulating substrates. Importantly, this click chemistry approach was amenable to widely utilized microfabrication approaches such as micropatterning and wet spinning. This study highlights a novel approach to prepare human protein‐based hydrogels that is well‐suited for scale‐up and could potentially be used as scaffolds for a variety of keratin‐rich tissues.

DOI: 10.1002/btm2.10077

### A portable laboratory

Low volume and portable testing of patient blood samples could address unmet needs in both the developed and developing world, such as rapid benchside diagnostics and blood‐testing for patients who do not have access to large clinical labs. One of the main challenges with the existing portable devices is the lack of detection of analytes via multiple analytical methods (e.g. ELISA, molecular diagnostics, etc.) at low blood volumes. This limits the use of these devices thereby promoting continued reliance on large clinical labs. Described in this issue of Bioengineering & Translational Medicine, a team from Theranos details the design and implementation of a clinical laboratory system (miniLab) that is portable, minimally designed (3 component system), and capable of detecting analytes in blood via a variety of detection methods ranging from molecular diagnostics to immunoassays. Specifically, the miniLab showed capability to analyze blood samples for both: (i) detection of Zika virus via molecular diagnostics and HSV‐2 via immunoassay, and for (ii) descriptive clinical panels for lipids and lymphocytes. This work describes a single, tunable, device that is capable of providing a means to test for multiple clinically‐relevant diseases and provide descriptive analysis for essential blood panels.

DOI: 10.1111/btm2.10084

## Recent literature

### A new role for nanoparticle therapeutics

Research from the Ashutosh Chilkoti lab from the Department of Biomedical Engineering at Duke University describes how a nanoparticle‐based approach to treat tumors can facilitate additional anti‐tumor benefits by modulating the tumor microenvironment towards improved antitumor immune response. This work focused on utilizing a previously described nanoparticle system that leverages chimeric polypeptides to self‐assemble into nanoparticles in the presence of doxorubicin. The authors show that an injection of their nanoparticle chimeric polypetide doxorubicin formulation induces a significant (nearly 2‐fold more) increase in the number of tumor‐infiltrating leukocytes, as compared to free doxorubicin. Specifically, the nanoparticle formulation stimulated CD8+ T cells and increased the ratio of Th1 to Th2 cytokines in the tumor, which are both antitumor immunomodulatory effects. The authors postulate that the cause for this is likely due to increased drug accumulation at the tumor, to the point where the immunomodulatory effects of the drugs can be achieved. It is possible that nanoparticle delivery systems can benefit from these findings, so that they are better designed and poised for clinical treatment.

*Mastria et al., Journal of Controlled Release. 2017; doi:* 10.1016/j.jconrel.2017.11.021

